# STAT3‐Mutated Hyper‐IgE Syndrome With Retroperitoneal Abscess in Adolescence

**DOI:** 10.1002/ccr3.71980

**Published:** 2026-02-04

**Authors:** Hiroaki Sugiyama, Yousuke Higuchi, Shintaro Fujiwara, Koki Aya, Wataru Mukai, Takafumi Goto

**Affiliations:** ^1^ Department of Pediatrics NHO Okayama Medical Center Okayama Japan; ^2^ Department of Pediatric Surgery NHO Okayama Medical Center Okayama Japan

**Keywords:** hyper‐IgE syndrome, methicillin‐sensitive *Staphylococcus aureus*, primary immunodeficiency disorder, retroperitoneal abscess, *STAT3* variant

## Abstract

Hyperimmunoglobulin E syndrome (HIES) is a rare primary immunodeficiency disorder characterized by eczema, recurrent staphylococcal infections, and significantly elevated serum IgE levels. An 18‐year‐old female presented with acute abdominal pain and was diagnosed with a retroperitoneal abscess. She had a history of recurrent skin abscesses, otitis media, and eczema since infancy, skeletal fractures, and retained primary teeth. Laboratory findings showed a serum IgE level above 20,150 U/L and a CRP of 180.30 mg/L. Methicillin‐sensitive *Staphylococcus aureus* was cultured from the abscess drainage. The NIH‐HIES score was 60 points. Genetic testing identified a heterozygous *STAT3* variant (NM_139276.3: c.1145G>A, p.(Arg382Gln)), confirming autosomal‐dominant HIES. This rare clinical presentation emphasizes the importance of considering HIES, even when deep‐seated infections develop outside typical cutaneous or pulmonary sites.

## Key Clinical Message

Clinicians should recognize that HIES can remain undiagnosed until adulthood. Identifying key features such as recurrent *Staphylococcus aureus* infections, eczema, markedly elevated IgE levels, and skeletal or dental anomalies is crucial for timely diagnosis and effective treatment, even in rare cases such as deep‐seated retroperitoneal abscesses.

## 1 Introduction

Hyperimmunoglobulin E syndrome (HIES) is a primary immunodeficiency disorder characterized by recurrent skin and lung infections, eczematous dermatitis, and elevated serum IgE levels. HIES was initially described as Job's syndrome by Davis et al. in 1966 [1] and was later defined immunologically by Buckley et al. in 1972 [2]. Although infections are usually limited to the skin and lungs, involvement of other areas may occasionally occur [3]. This report describes a case of HIES complicated by a retroperitoneal abscess.

## 2 Case History/Examination

**FIGURE 1:**
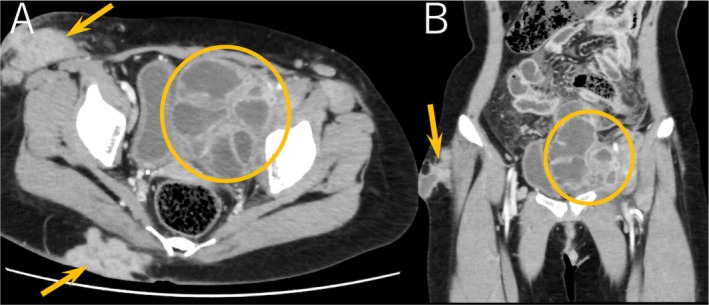
Contrast‐enhanced abdominal CT (A: axial view, B: coronal view) scan shows a 10.5 cm multilocular retroperitoneal abscess with rim enhancement extending from the pelvis to the left inguinal area (circle). Arrows indicate scars from cutaneous abscesses.

An 18‐year‐old woman with no significant family history presented to the emergency department with a sudden onset of lower abdominal pain. She exhibited a high fever and severe midline lower abdominal tenderness with a guarding reflex. Chronic inflammatory changes resulted in mass‐like lesions extending from the lower abdomen to the groin. Laboratory tests revealed elevated CRP (180.30 mg/L), a normal white blood cell count (6.00 × 10^9^/L), and significantly elevated serum IgE levels (> 20,150 U/L). Abdominal contrast‐enhanced computed tomography (CT) indicated a 10.5 cm multilocular retroperitoneal abscess (Figure 1). Ultrasound‐guided aspiration revealed gram‐positive cocci.

## 3 Differential Diagnosis

She had a history of recurrent cutaneous abscesses since childhood, affecting the face, abdomen, and groin, and methicillin‐sensitive *Staphylococcus aureus* (MSSA) was consistently isolated from the abscesses. Each episode was treated using incision, drainage, or antibiotic therapy. MSSA was identified as the causative organism, and treatment with cefazolin was initiated. Subsequently, MSSA was cultured from abscess drainage.

Further history revealed that the patient had had severe facial eczema since 2 days of age, multiple fractures during infancy, recurrent otitis media, and retention of four primary teeth at 13 years of age. Physical examination showed characteristic facial features, including a mildly prominent forehead and broad nasal bridge. Submucous cleft palate and high‐arched palate were also observed. Chest X‐ray revealed mild scoliosis, but no evident joint hyperextensibility. In addition, markedly elevated serum IgE levels (> 20,000 IU/mL) were observed. These findings had previously raised the suspicion of HIES; however, genetic testing was not performed at that time due to limited access to tests.

## 4 Conclusion and Results

**FIGURE 2:**
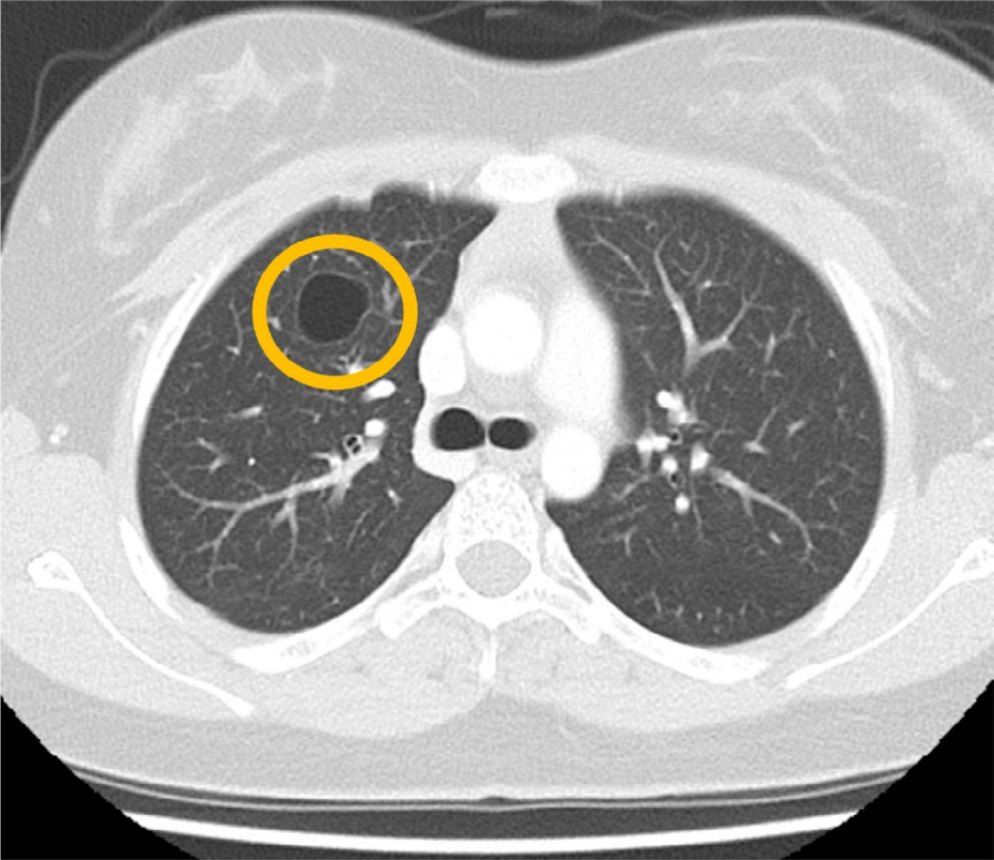
Chest CT reveals a pulmonary cyst in the right upper lobe (circle).

Later, chest CT performed after a traffic accident revealed pulmonary cysts (Figure 2). With these additional pulmonary findings, the patient's NIH‐HIES score reached 60, strongly suggesting HIES (Table 1) [4]. Genetic testing was performed at Kazusa DNA Research Institute using a targeted gene panel for HIES that includes the following genes: *STAT3, TYK2, IL6R, ZNF341, ERBIN, TGFBR1, TGFBR2, SPINK5, PGM3, CARD11*, and *DOCK8*. A heterozygous pathogenic variant was identified in the DNA‐binding domain of the *STAT3* gene (GRCh38, NM_139276.3: c.1145G>A p.(Arg382Gln); ClinVar accession VCV000018305.48). Although segregation analysis was not performed, we confirmed the diagnosis of autosomal‐dominant HIES (AD‐HIES) [5, 6]. The retroperitoneal abscess was resolved with aspiration and antibiotic therapy, and the patient was discharged on Day 14. Prophylactic trimethoprim‐sulfamethoxazole was initiated.

**TABLE 1:** NIH‐HIES Scoring System: The relevant findings for the patient are indicated in bold.

Clinical findings	Points									
	0	1	2	3	4	5	6	7	8	10
Highest serum‐IgE level (IU/mL)	< 200	200–500			501–1000				1001–2000	**≥ 2000**
Skin abscesses	None		1–2		3–4				**≥ 4**	
Pneumonia (episodes over lifetime)	**None**		1		2		3		> 3	
Parenchymal lung anomalies	Absent						Bronchiectasis		**Pneumatocele**	
Retained primary teeth	**None**	1	2		3				> 3	
Scoliosis, maximum curvature	< 10°		**10°–14°**		15°–20°				> 20°	
Fractures with minor trauma	None			1–2					**> 2**	
Highest eosinophil count (cells/ml)	**< 700**			700–800			> 800			
Characteristic face	Absent			Mildly present		**Present**				
Midline anomaly	Absent					**Present**				
Newborn rash	Absent				**Present**					
Eczema (worst stage)	Absent	Mild	Moderate		**Severe**					
Upper respiratory infections/year	**1–2**	3	4–6		> 6					
Candidiasis	**None**	Oral	Fingernails		Systemic					
Other serious infections	None				**Severe**					
Fatal infection	**Absent**				Present					
Hyperextensibility	**Absent**				Present					
Lymphoma	**Absent**				Present					
Increased nasal width	**< 1 SD**	1–2 SD		> 2 SD						
High palate	Absent		**Present**							
Young‐age correction	**≥ 5 years**			2–5 years		1–2 years		< 1 year		

## 5 Discussion

HIES is characterized by elevated serum IgE levels, chronic eczematous dermatitis with cold abscesses, recurrent infections, such as pneumonia and otitis media, delayed tooth eruption, skeletal fractures, distinctive facial features, eosinophilia, and impaired neutrophil chemotaxis.

Although high serum IgE levels are hallmarks, they are not a central pathophysiological mechanism. Instead, mutations in the *STAT3* gene disrupt the JAK–STAT signaling pathway, particularly by impairing Th17 cell differentiation. This disruption leads to an increased susceptibility to staphylococcal infections, atopic dermatitis, skeletal and joint abnormalities, and elevated IgE levels [7]. Immune dysregulation resulting from Th17 deficiency induces a compensatory Th2‐dominant immune response that contributes to the development of hyper‐IgE.

Variants impacting residue Arg382 in the STAT3 DNA‐binding domain—most commonly Arg382Gln and Arg382Trp—are recurring hotspots in AD‐HIES [7, 8]. These substitutions usually maintain STAT3 protein phosphorylation and dimerization but impair DNA binding and transactivation, exerting dominant‐negative effects and leading to significant Th17 deficiency and the characteristic AD‐HIES phenotype [9]. Furthermore, *STAT3* variants exhibit functional diversity; gain‐of‐function mutations cause infantile‐onset multisystem autoimmune disease‐1 (ADMIO1). This functional split indicates that STAT3 signaling is key to both immune deficiency and autoimmunity [10].

Several reports have documented that a definitive diagnosis of HIES may take several years after the initial onset of symptoms. Cases associated with *STAT3* variants are often not diagnosed until adulthood. An Italian registry study of 30 patients with autosomal dominant HIES reported a mean age at symptom onset of 12 months, with 66.7% of patients presenting before the age of 1 year. Among those with early onset disease, the median age at diagnosis was 12.1 years. The mean diagnostic delay was 13.7 ± 13.2 years [11]. In this cohort, 76.7% of the patients had a history of pneumonia, 43.3% had pneumothorax at diagnosis, 46.7% had bronchiectasis, and 13.3% developed invasive pulmonary mycoses.

Diagnosis often depends on recognizing characteristic clinical signs and symptoms. However, the rarity of HIES and its features overlapping with common atopic conditions may hinder its recognition. The presence of cold abscesses is a key feature of this condition. In STAT3‐deficient mice, hepatic acute‐phase responses mediated by IL‐6 are impaired, resulting in minimal initial inflammatory symptoms. IL‐6, rather than CRP, may serve as a more useful marker for early infection detection [6, 7]. The misinterpretation of eczema and elevated IgE levels may also lead to diagnostic delays [11].

Recurrent skin abscesses and pneumonia are key clinical features for diagnosing HIES. A USIDNET‐based analysis of 85 patients with autosomal dominant HIES born between 1950 and 2013 revealed that 74.4% had skin abscesses and 72% had pneumonia [12]. Although infections primarily affect the skin and lungs, deep‐seated infections can also occur. One report described a 5‐year‐old boy with AD‐HIES who developed multiple pelvic abscesses, leading to obstructive uropathy and postrenal failure [13]. In our case, the retroperitoneal abscess was likely caused by the same organism responsible for the previous superficial infections.

STAT3 also regulates matrix metalloproteinases (MMPs), and abnormal MMP levels have been documented in patients with STAT3 protein deficiency [14]. MMPs are proteolytic enzymes that remodel the extracellular matrix and contribute to wound healing and angiogenesis [15]. In murine models, STAT3 protein deficiency impairs mucosal healing processes. One patient with colonic perforation had multiple ulcers at the perforation site [14]. Th17 cells also help maintain the integrity of the gut mucosal barrier, and their deficiency may contribute to gastrointestinal infections and perforations, which could lead to retroperitoneal abscess formation. Although our case involved an extraperitoneal abscess, the possibility of a GI‐related cause in HIES should be considered when evaluating patients with abdominal symptoms and systemic signs suggestive of HIES.

Clinicians should recognize that HIES can remain undiagnosed until adulthood. Identifying characteristic features such as recurrent Staphylococcus infections, eczema, markedly elevated IgE levels, and skeletal or dental anomalies is crucial for timely diagnosis and effective management, even in atypical presentations like deep‐seated retroperitoneal abscesses.

## Author Contributions

**Yousuke Higuchi:** conceptualization, supervision, writing – review and editing. **Hiroaki Sugiyama:** conceptualization, data curation, writing – original draft. **Shintaro Fujiwara:** data curation, writing – review and editing. **Koki Aya:** data curation, writing – review and editing. **Wataru Mukai:** data curation, investigation, writing – review and editing. **Takafumi Goto:** conceptualization, investigation, writing – review and editing.

## Funding

The authors have nothing to report.

## Ethics Statement

All procedures performed in this study were conducted in accordance with the ethical standards of the NHO Okayama Medical Center and with the 1964 Declaration of Helsinki and its later amendments.

## Consent

Written informed consent was obtained from the patient and her parents for genetic testing and publication of this case report, along with any accompanying images.

## Conflicts of Interest

The authors declare no conflicts of interest.

## Data Availability

The data that support the findings of this study are available from the corresponding author upon reasonable request.
